# Effectiveness of virtual reality in reducing anxiety, fear, and pain in children undergoing skin prick testing: A crossover study

**DOI:** 10.1111/pai.70235

**Published:** 2025-11-01

**Authors:** Rita Nocerino, Adriana Napolitano, Giorgio Bedogni, Teresa Rea, Silvio Simeone, Antonio Masino, Erika Caldaria, Serena Coppola, Laura Carucci, Roberto Berni Canani

**Affiliations:** ^1^ Department of Translational Medical Science University of Naples “Federico II” Naples Italy; ^2^ NutriTechLab, University of Naples “Federico II” Naples Italy; ^3^ Department of Biomedicine and Prevention University of Rome “Tor Vergata” Rome Italy; ^4^ University of Naples “Federico II” Naples Italy; ^5^ Department of Medical and Surgical Sciences Alma Mater Studiorum‐University of Bologna Bologna Italy; ^6^ Department of Primary Health Care Internal Medicine Unit Addressed to Frailty and Aging, “S. Maria Delle Croci” Hospital, AUSL Romagna Ravenna Italy; ^7^ Department of Public Health University of Naples “Federico II” Naples Italy; ^8^ Department of Clinical and Experimental Medicine University of Catanzaro Magna Graecia Catanzaro Italy; ^9^ European Laboratory for the Investigation of Food‐Induced Diseases, University of Naples “Federico II” Naples Italy; ^10^ Task Force for Microbiome Studies, University of Naples “Federico II” Naples Italy

**Keywords:** allergy screening tests, anxiety, children, compliance, non‐pharmacological interventions, pain, skin prick test, virtual reality

## Abstract

**Background:**

Skin prick testing (SPT) is a cornerstone diagnostic procedure for identifying allergic sensitizations in children. Despite being minimally invasive, it often provokes considerable anxiety, fear, and pain, potentially compromising test accuracy due to poor compliance. Virtual reality (VR) has emerged as a promising non‐pharmacological tool for procedural distress management, yet its application in allergy diagnostics remains underexplored.

**Objective:**

To evaluate the effectiveness of VR in reducing procedural anxiety, fear, and pain, and improving compliance in children undergoing SPT.

**Methods:**

This single‐center, cross‐over interventional study enrolled 108 children (aged 4–18 years) with suspected or confirmed environmental or food allergies. Each participant underwent two SPT sessions: one with immersive VR distraction and one using standard‐of‐care (SOC) distraction methods, separated by a 6‐month washout period. Outcomes were assessed using validated scales for anxiety (Children's Anxiety Meter), fear (Children's Fear Scale), pain (Wong‐Baker FACES), and procedural compliance (modified Induction Compliance Checklist). Physiological parameters and staff satisfaction were also recorded.

**Results:**

VR significantly reduced anxiety, fear, and pain compared to SOC across multiple time points, with marked improvements in compliance (100% full compliance in the VR group vs. 0% in SOC) and staff satisfaction. No adverse events were reported, confirming the safety of VR in this setting.

**Conclusions:**

VR is a safe and effective tool for minimizing procedural distress and enhancing compliance in children undergoing SPT. Its integration into routine allergy diagnostics may improve patient experience and procedural success.

**Abbreviations**
BP: blood pressure
CAM: children's anxiety meter
CFS: Children's Fear Scale
CRFs: case report forms
DBP: diastolic blood pressure
EAACI: European Academy of Allergy and Clinical Immunology
HR: heart rate
IQR: interquartile range
mICC: Modified Induction Compliance Checklist
ORCID: Open Researcher and Contributor ID
RELR: random effects linear regression
SBP: systolic blood pressure
SOC: standard of care
SpO_2_
: oxygen saturation
SPT: skin prick test
VAS: visual analog scale
VR: virtual reality

**Key message**

This study demonstrated that the use of virtual reality during skin prick testing significantly reduces procedural anxiety, fear, and pain in children, while markedly improving compliance and staff satisfaction. These findings support the integration of immersive virtual tools as safe and effective adjuncts in routine pediatric allergy diagnostics to enhance patient experience and procedural success.

## 1 INTRODUCTION

Allergic diseases are a growing global health concern, affecting millions of individuals worldwide, with an increasing prevalence in pediatric populations.^1^, ^2^ According to the European Academy of Allergy and Clinical Immunology (EAACI) Advocacy Manifesto, over 150 million Europeans currently suffer from chronic allergic diseases, with projections indicating that by 2025, more than 50% of the European population will be affected. Specifically, the document states that 100 million Europeans suffer from allergic rhinitis and 70 million from asthma.^3^

The skin prick test (SPT) is one of the most widely used diagnostic tools for identifying immediate hypersensitivity reactions and is frequently performed in allergy clinics to detect sensitization to various allergens.^4^ This test involves the application of small amounts of allergens on the skin, followed by pricking to facilitate antigen penetration. While generally safe and minimally invasive, the procedure often induces anxiety, fear, and discomfort in children, leading to compliance issues and, in some cases, test failure.^5^

Although generally considered minimally invasive, the SPT may still cause significant anxiety, fear, and pain in children—especially in those who are younger, more sensitive, or have had previous negative experiences with medical procedures—potentially affecting both their compliance and the accuracy of test results.^5^, ^6^, ^7^

Although exact statistics are not readily available, market‐based estimations suggest that approximately 5.4 million SPTs are performed annually in Europe, assuming an average test cost of $50 and a European market share of 48.5% of the global allergy skin test market, valued at $560 million in 2022.^8^

However, compliance remains a significant challenge, with up to 39% of children failing to complete the SPT properly due to discomfort or distress during the procedure.^9^

This noncompliance and distress during SPT can compromise diagnostic accuracy, leading to the need for repeat testing, prolonged medical consultations, and increased direct and indirect healthcare costs.^10^ Missed or delayed diagnoses due to test failure can have severe consequences, as undiagnosed allergic conditions can lead to unnecessary medication use, uncontrolled symptoms, and a higher risk of severe allergic reactions, particularly in children with asthma or food allergies.^11^, ^12^ Additionally, patients must discontinue antihistamines and corticosteroids at least 7 days prior to testing, further complicating management if the test fails. On the other hand, unrecognized allergies can lead to mismanagement of symptoms, potentially resulting in increased healthcare visits and higher costs for emergency care.^13^

To improve patient compliance and minimize distress, various distraction techniques have been explored, including parental presence, music therapy, storytelling, and toys.^14^, ^15^, ^16^ While these interventions have some effectiveness, they often fail to fully distract highly anxious children or those with previous negative medical experiences.^6^ As a result, alternative interactive distraction techniques have gained attention as potential solutions.

Virtual reality (VR) has emerged as a promising tool in pediatric pain management.^16^ Unlike passive distractions such as watching cartoons, VR provides an immersive, interactive environment that can effectively capture a child's attention, reducing their perception of pain and distress.^6^ The Multiple Resource Theory suggests that VR redirects attentional resources away from nociceptive stimuli, thereby diminishing pain perception and anxiety.^7^ Several studies have demonstrated that VR can reduce pain intensity by up to 50%, and anxiety by over 30%, in children undergoing medical procedures such as venipuncture, burn care, and vaccinations.^5^, ^16^ Furthermore, a systematic review found that VR is particularly effective in children aged 5–10 years for managing preoperative anxiety, supporting its broader applicability in pediatric healthcare.^17^

Despite its established efficacy in other pediatric procedures, limited research has examined the role of VR during SPT.^5^ However, emerging evidence from other medical disciplines highlights VR as a viable alternative to pharmacological interventions for pain and anxiety reduction. For instance, VR has been found comparable to nitrous oxide in reducing pain and anxiety during minor surgical procedures in children, and its use in pediatric dentistry has led to a 54% reduction in anxiety and a 50% decrease in pain perception.^18^, ^19^ Additionally, a recent pilot study demonstrated that VR goggles approved for infectious disease control effectively lowered fear and anxiety in common pediatric procedures, such as venipunctures and drain removals.^20^

Based on this evidence, this study aims to evaluate the effectiveness of VR in reducing pain, fear, and anxiety in children undergoing SPT, using a crossover design. Patients were randomized into either a VR or Standard of Care (SOC) group, with the intervention repeated after 6 months, switching the groups.

Additionally, this study also examines physiological parameters (heart rate, oxygen saturation, and blood pressure), compliance rate, and staff satisfaction to assess the broader clinical implications of VR implementation. By addressing these factors, we aim to provide evidence‐based recommendations for integrating VR as a standard adjunct in pediatric allergy testing, ultimately enhancing patient cooperation, reducing procedural failure rates, and improving healthcare efficiency.

## 2 METHODS

### 2.1 Study design and study population

This study was a single‐center, non‐pharmacological interventional trial with a crossover design. The study included children aged 4–18 years with suspected or diagnosed allergies to environmental or food allergens. Participants were recruited from the Pediatric Allergy Clinic of the University of Naples Federico II, where they were referred for routine skin prick testing. Written informed consent was obtained from parents or legal guardians, and assent was provided by children aged ≥6 years.

The decision to adopt a crossover design was made to allow each child to serve as their own control, thereby reducing variability and increasing statistical power in comparing VR versus SOC conditions. Children underwent two SPT sessions spaced 6 months apart, one using VR and the other using SOC distraction techniques, as part of the routine clinical follow‐up protocol for food and environmental allergy diagnosis.

Inclusion criteria required participants to have a confirmed history of allergic symptoms related to either environmental or food allergens and to be between 4 and 18 years of age. Patients were excluded if they had a history of seizure disorders, motion sickness, or severe developmental delay, as these conditions could interfere with the use of VR. Additionally, non‐Italian‐speaking patients were excluded to ensure proper communication and understanding of study procedures. Participants who had used systemic antihistamines or corticosteroids within the past 7 days were also excluded to avoid interference with the SPT results.

### 2.2 Randomization and intervention

Randomization was performed using a computer‐generated sequence, assigning participants to either the VR intervention group or the SOC group for their initial test session. After a six‐month washout period, participants crossed over to the alternate group during their follow‐up SPT appointment. In the VR group, participants used an interactive VR application via a head‐mounted display 1 min before and throughout the SPT procedure. The VR intervention utilized a Samsung Gear VR headset (Samsung, Seoul, South Korea) in conjunction with a Samsung S7 or S8 mobile device to provide an immersive and engaging experience. Children were allowed to choose from a range of age‐appropriate VR content designed to enhance engagement and relaxation. Prior to the procedure, children were trained on how to use the headset by study staff to ensure a smooth experience. The SOC group received traditional distraction techniques, including access to toys and parental reassurance.

### 2.3 Ethics

The study protocol, patient information sheet, informed consent form, and clinical chart were reviewed and finally approved by the Territorial Ethical Committee of the University of Naples Federico II with number 198. The study adhered to the Helsinki Declaration (Helsinki revision, 2024), Good Clinical Practice standards (CPMP/ICH/135/95), and relevant European and Italian data protection regulations. This study was registered in the Clinical Trials Protocol Registration System with the ID number NCT06952192.

### 2.4 Data collection

During the initial visit, experienced pediatricians evaluated each subject for eligibility. Demographic and medical history data were collected.

After obtaining informed consent from parents or legal guardians, data collection was conducted at three time points: before (pre), during (during), and 1 min after (post) the SPT procedure, which was performed either with or without VR according to the randomization list. Specifically, pre measurements were collected after VR immersion had started (approximately 1 min before the procedure), but immediately prior to skin contact with the lancet, allowing us to capture the anticipatory effect of the intervention. In the SOC group, the same timing was maintained, with standard distraction techniques already in place.

Before the test, each child received a brief, age‐appropriate explanation of the SPT procedure from the nurse, including a description of the steps and visual presentation of the lancet device. No test prick or simulation was performed prior to the actual procedure in order to minimize anticipatory sensitization. This pretest interaction was standardized across all participants.

SPT was performed on the volar surface of the forearm using commercially available standardized allergen extracts (ALK‐Abelló, Hørsholm, Denmark). A 1‐mm single‐peak sterile lancet (ALK) was used to apply the allergens perpendicularly through a drop of extract. Each child was tested with a panel of up to 18 allergens, including food (i.e., milk, egg, peanut, tree nuts, wheat, soy, fish, and shellfish) and aeroallergens (i.e., dust mite, grass pollens, tree pollens, molds, cat, dog, and cockroach), depending on the clinical history. Histamine dihydrochloride (10 mg/mL) and isotonic saline solution (NaCl 0.9%) served as positive and negative controls, respectively. The distance between adjacent test sites was at least 2 cm to avoid overlapping reactions. After 15 min, the wheal and flare reaction was measured using a transparent millimeter ruler, and the largest wheal diameter was recorded. A test was considered positive when the wheal diameter was ≥3 mm in the absence of a reaction to the negative control.^21^ All SPTs were performed by two experienced pediatric nurses, each with over 5 years of clinical practice in allergy diagnostics. Prior to study initiation, both nurses received standardized training to ensure procedural consistency. This training included a review of SPT procedures, hands‐on demonstrations, and supervised practice under a senior pediatric allergist. The same team performed all tests throughout the study period, and no inter‐operator variability in execution was observed.

Procedural distress was assessed through validated instruments appropriate for the child's age and developmental level. Anxiety was measured using the Children's Anxiety Meter (CAM), a vertical analog scale ranging from 0 (completely calm) to 10 (extremely anxious).^22^ Fear was assessed using the Children's Fear Scale (CFS), a pictorial tool representing escalating facial expressions of fear.^23^ Pain perception was measured with the Wong‐Baker FACES Pain Rating Scale, ranging from 0 (no pain) to 10 (worst pain) with corresponding facial expressions.^24^, ^25^

Compliance was assessed using the Induction Compliance Checklist (mICC), a 10‐item observational checklist measuring behaviors interfering with the procedure, such as crying, verbal refusal, and physical resistance. Higher scores on the mICC indicated lower compliance.^26^ Physiological parameters, including heart rate (HR), oxygen saturation (SpO_2_), and blood pressure (BP), were recorded before and after the test using a pulse oximeter for HR and SpO_2_ and a manual sphygmomanometer for BP.

Finally, staff satisfaction was measured using the Staff Satisfaction Scale, an 8‐item questionnaire where healthcare providers rated aspects such as the child's understanding, cooperation, and emotional needs on a scale from 1 (strongly disagree) to 5 (strongly agree).^27^

The incidence of adverse events, such as dizziness or nausea related to VR use, was monitored throughout the study.

### 2.5 Data entry

Data were recorded anonymously in case report forms (CRFs). Completeness and accuracy were verified by two researchers. Data were entered into a secure database and reviewed by a biostatistician for data cleaning and analysis before database locking.

### 2.6 Study outcomes

The primary objective of this study was to evaluate the effectiveness of VR in reducing procedural anxiety in children undergoing SPT. Anxiety levels were assessed using the CAM scale pre, during, and post procedure, that is, 1 min after the procedure.

Secondary outcomes included the evaluation of pain, fear, and procedural compliance before, during, and 1 min after the procedure.

Additional secondary outcomes included the assessment of physiological parameters (HR, SpO_2_, and BP) before and after the procedure.

Finally, adverse events, including dizziness, nausea, or discomfort associated with VR use, were monitored throughout the study.

### 2.7 Sample size

Given the exploratory nature of the study and the lack of prior data for effect size estimation in this specific setting, a formal power calculation was not performed. The sample size was determined based on feasibility and alignment with prior VR studies in pediatric procedural care. The effect size estimates obtained from this study will be used to inform sample size calculations for future adequately powered randomized controlled trials.

### 2.8 Statistical analysis

Continuous variables are reported as median (50th percentile) and interquartile range (IQR, difference between the 75th and 25th percentiles). Discrete variables are reported as the number and proportion of participants with the characteristic of interest. The main crossover analysis of the outcomes under VR and SOC was performed using random effects linear regression (RELR). The predictors of RELR were the procedure (discrete: 0 = SOC; 1 = VR), time (discrete: 0 = time 0; 1 = time 1; 2 = time 2), a procedureXtime (discreteXdiscrete) interaction, and the sequence of the procedure (0 = SOC‐VR; 1 = VR‐SOC).^28^, ^29^ The inclusion of the sequence variable (VR‐first vs. SOC‐first) allowed us to test whether maturation or order effects influenced the outcomes across the 6‐month interval. The random effect was assigned to the child, and robust confidence intervals were used to relax the homoscedasticity assumption made by RELR.^28^ The between‐procedure within‐time values of the outcomes and their differences were calculated as marginal probabilities with a Bonferroni correction for multiple comparisons (contrasts).^28^, ^30^ To evaluate the effect of age (continuous) at enrollment on the treatment × time interaction (discrete × discrete), a multivariable RELR model was fitted, including the 3‐way interaction procedure × time × age (discrete × discrete × continuous) with all relevant main effects and interactions as predictors.^28^, ^30^, ^31^ Statistical analysis was performed using Stata 19.5 (Stata Corporation, College Station, TX, USA) and using SPSS 23.0 (IBM Corporation, Armonk, NY, USA). Statistical significance was set at *p* < .05 for all analyses.

## 3 RESULTS

### 3.1 Study population

From February 2024 to February 2025, 110 consecutive children were evaluated for participation in the study. Of these, two children refused to participate. Consequently, a total of 108 children were enrolled in the study, and all of them completed both phases of the crossover study. Each participant underwent two testing sessions, one under the VR procedure and the other under the SOC procedure with a 6‐month washout period between sessions. Table 1 summarizes the main demographic and clinical characteristics of the study population. No child had ever used VR before enrolling in the study.

**TABLE 1:** Baseline characteristics of study population at enrollment.

*N*	108
Sex	
Female	35 (32.4%)
Male	73 (67.6%)
Age (months)	92.0 (64.0)
Age group distribution	
4–5 year	13 (12%)
6–12 year	73 (67.6%)
13–18 year	22 (20.4%)
Delivery	
Normal	33 (30.6%)
Cesarean	75 (69.4%)
Gestational age (months)	40.0 (0.0)
Birth weight (g)	3130.0 (685.0)
Age at weaning (months)	5.0 (1.0)
Passive smoking	
No	64 (59.3%)
Yes	44 (40.7%)
Mother smoke during pregnancy	
No	69 (63.9%)
Yes	39 (36.1%)
House	
Urban	75 (69.4%)
Rural	33 (30.6%)
Pets	
No	84 (77.8%)
Yes	24 (22.2%)
Siblings	
No	19 (17.6%)
Yes	89 (82.4%)
Siblings (number)	1.0 (1.0)
Family risk of allergy	
No	30 (27.8%)
Yes	78 (72.2%)
Family members with allergy	1.0 (0.0)
Food allergy	
No	80 (74.1%)
Yes	28 (25.9%)
Environmental allergy	
No	13 (12.0%)
Yes	95 (88.0%)
Skin symptoms	
No	64 (59.3%)
Yes	44 (40.7%)
Respiratory symptoms	
No	22 (20.4%)
Yes	86 (79.6%)
Gastrointestinal symptoms	
No	93 (86.1%)
Yes	15 (13.9%)
Anaphylaxis	
No	80 (74.1%)
Yes	28 (25.9%)
Number of skin prick test performed	16 (0.0)
Weight (kg)	29.5 (28.7)
Weight (SDS WHO)	0.8 (2.0)
Height (m)	127.7 (32.2)
Height (SDS WHO)	0.2 (1.5)
BMI (kg/m^2^)	18.6 (6.7)
BMI (SDS WHO)	1.0 (1.9)

*Note*: Values are median (IQR) for continuous variables and number (percentage) for discrete variables.

### 3.2 Main study outcome

**FIGURE 1:**
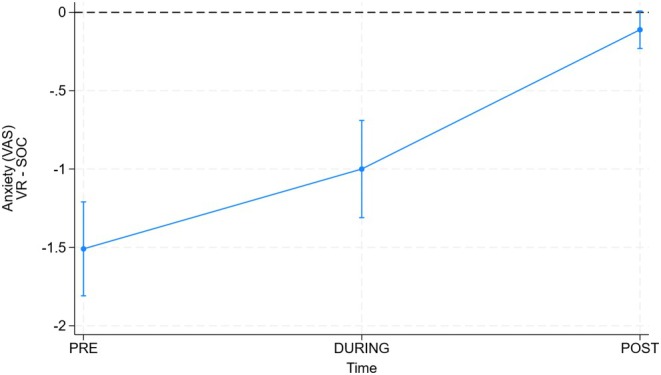
Difference in anxiety (main outcome) between the VR and SOC procedures before (PRE), during (DURING), and after (POST) session. Values are obtained by random‐effects linear regression (see statistical analysis for details). 95% CI is corrected for 3 (times) comparisons using Bonferroni correction.

The results of the primary study outcome, that is, anxiety level, are reported in Table 2 and in Figure 1. As estimated by the RELR model (not shown), the use of VR was associated with lower values of anxiety at pre‐, during‐, and post‐times. Such difference was statistically and clinically relevant at times pre and during but not at time post. Importantly, the sequence of the procedures (VR‐first vs. SOC‐first) did not significantly affect anxiety scores, indicating that maturation or order effects over the six‐month interval did not bias the results.

**TABLE 2:** The between‐procedure difference in the outcomes of interest as estimated by RELR.

Outcome	SOC			VR			Δ (VR – SOC)		
	Pre	During	Post	Pre	During	Post	Pre	During	Post
Anxiety (VAS)^a^	3.5 (3.0 to 4.1)	2.4 (1.9 to 2.9)	1.2 (1.0 to 1.5)	2.0 (1.7 to 2.4)	1.4 (1.1 to 1.7)	1.1 (0.9 to 1.3)	−1.5 (−1.8 to −1.2) *p* < .001	−1.0 (−1.3 to −0.7) *p* < .001	−0.1 (−0.2 to 0.01) *p* = .077
Pain (VAS)^b^	—	3.6 (3.0 to 4.1)	0.4 (0.1 to 0.6)	—	1.9 (1.4 to 2.3)	0.1 (0.01 to 0.2)	—	−1.7 (−2.0 to −1.5) *p* < .001	−0.2 (−0.4 to −0.1) *p* = .004
Fear (VAS)^a^	1.4 (1.1–1.7)	0.8 (0.5 to 1.0)	0.1 (0.01 to 0.1)	0.7 (0.4–0.9)	0.2 (0.1 to 0.4)	0.01 (−0.01 to 0.01)	−0.8 (−1.0 to −0.6) *p* < .001	−0.5 (−0.7 to −0.3) *p* < .001	−0.1 (−0.1 to 0.01) *p* = .09
Heart rate (BPM)^b^	—	82 (79 to 85)	91 (88 to 94)	—	82 (79 to 85)	88 (85 to 91)	—	0.1 (−0.2 to 0.4) *p* = .818	−2.4 (−3.3 to −1.4) *p* < .001
SpO_2_ (%)^b^	—	100 (99 to 100)	100 (99 to 100)	—	100 (99 to 100)	100 (99 to 100)	—	0.0 (−0.1 to 0.2) *p* = .987	0.0 (−0.1 to 0.1) *p* = 1.000
SBP (mm Hg)^b^	—	105 (103 to 108)	106 (104 to 109)	—	105 (102 to 107)	104 (101 to 106)	—	−0.6 (−1.3 to −0.01) *p* = .034	−2.6 (−3.7 to −1.5) *p* < .001
DBP (mm Hg)^b^	—	72 (71 to 73)	72 (71 to 73)	—	72 (71 to 72)	71 (70 to 72)	—	−0.1 (−0.5 to 0.2) *p* = .635	−0.9 (−1.5 to −0.4) *p* < .001

*Note*: Values are means and robust 95% confidence intervals estimated from a random‐effects linear regression model with Bonferroni correction for multiple comparisons (see text for details).

^a^ *N* = 432.

^b^ *N* = 648 repeated measures.

**FIGURE 2:**
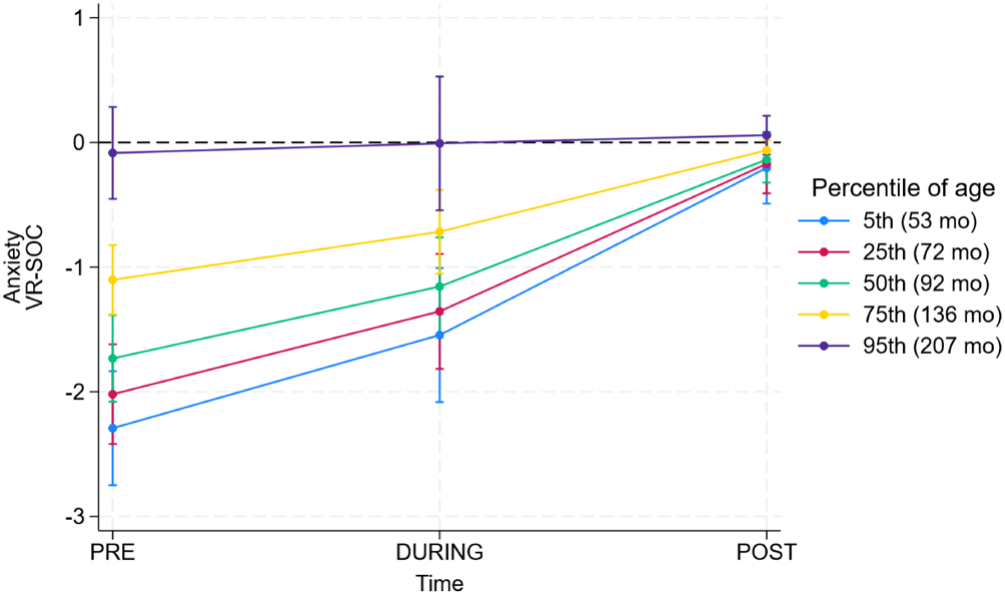
Difference in anxiety (main outcome) between the VR and SOC procedures before (PRE), during (DURING), and after (POST) session as a function of age at enrollment. Values from this secondary analysis are obtained by random‐effects linear regression with a 3‐way procedure × time × age interaction (see statistical analysis for details). 95% CI are corrected for 15 (3 times × 5 percentiles) comparisons using Bonferroni correction.

The Figure 2 plots the within‐time difference between VR and SOC as a function of continuous age as estimated by the RELR model including the procedure × time × age interaction (*p* < .0001 for the interaction, model not shown). Not unexpectedly, the largest differences between VR and SOC were seen at the 5th percentile of age at enrollment (53 months), and they progressively decreased with increasing percentile of age. Although this is an exploratory analysis which must therefore be taken with caution, there appears to be a decrease in the effectiveness of VR with increasing age. This is something that should be taken into account in the design of further RCTs on VR.

### 3.3 Secondary outcomes

The between‐procedure difference in the outcomes of interest as estimated by RELR is given in Table 2. Pain scores were significantly lower in the VR group compared to SOC, with a difference between groups of −1.7 (95% CI: −2.0 to −1.5; *p* < .001) at the first time point and −0.2 (95% CI: −0.4 to −0.1; *p* = .004) at the second.

The VR use also led to a significant reduction in perceived fear scores, with differences of −0.8 (95% CI: −1.0 to −0.6; *p* < .001) and −0.5 (95% CI: −0.7 to −0.3; *p* < .001) at the first and second time points, respectively, compared to SOC.

The HR was significantly lower in the VR group than in SOC at the second time point (Δpost = −2.4 bpm; 95% CI: −3.3 to −1.4; *p* < .001), with no difference observed at the first (Δduring = 0.1 bpm; 95% CI: −0.2 to 0.4; *p* = .818).

The SpO_2_ remained stable across groups, with no significant differences (Δduring = 0.0; *p* = .987; Δpost = 0.0; *p* = 1.000).

The systolic BP showed a slight but significant reduction in the VR group at the first time point (Δduring = −0.6 mmHg; 95% CI: −1.3 to −0.01; *p* = .034), and a more pronounced reduction at the second (Δpost = −2.6 mmHg; 95% CI: −3.7 to −1.5; *p* < .001).

The diastolic BP was significantly lower only at the second time point (Δpost = −0.9 mmHg; 95% CI: −1.5 to −0.4; *p* < .001), with no significant difference at the first (Δduring = −0.1 mmHg; 95% CI: −0.5 to 0.2; *p* = .635).

Compliance was 3.6 (95% CI 3.4–3.9) in the SOC versus 0.7 (0.5–1.0) in the VR group, corresponding to a significant difference of −2.9 (95% CI −3.1 to −2.7; *p* < .001, *n* = 216 repeated measures, RELR). In addition, 100% of patients in the VR group achieved full compliance, whereas none of the patients in the SOC group (0%) were fully compliant. Conversely, in the SOC group, 73% of patients were classified as noncompliant, compared to only 27% in the VR group. These significant findings suggest that the introduction of VR as a distraction technique substantially improved procedural adherence among children undergoing SPT.

Staff satisfaction was 18 (95% CI 17–18) in the SOC versus 38 (37–39) in the VR group, corresponding to a significant difference of 20 (95% CI 20–21; *p* < .001, *n* = 216 repeated measures, RELR). In addition, 100% of staff members in the SOC group reported dissatisfaction, whereas in the VR group, 55.6% of staff members reported being fully satisfied with the procedural experience. Conversely, the proportion of staff members who were not fully satisfied was substantially higher in the SOC group (69.2%) compared to the VR group (30.8%). These significant findings suggest that the introduction of VR not only improved patient cooperation but also contributed to a more positive experience for healthcare providers.

### 3.4 Safety

Throughout the study, no participants exhibited intolerance to the VR intervention or SOC procedure. Additionally, no adverse events were directly attributed to the use of VR or the standard procedural approach, ensuring that both interventions were well tolerated by all participants.

Adherence to the study protocol remained consistent across both groups, with no reported deviations that could compromise data integrity. Potential adverse events associated with VR use, including dizziness, nausea, or discomfort, were closely monitored throughout the study period. However, no participants reported experiencing significant side effects, further supporting the feasibility and safety of VR as a non‐pharmacological intervention for reducing procedural distress in children undergoing SPT.

## 4 DISCUSSION

The findings of this study confirm and extend previous literature supporting the use of VR as an effective non‐pharmacological intervention to manage procedural distress in children. Specifically, the implementation of VR during SPT resulted in significantly lower levels of anxiety, fear, and pain, as well as improved compliance and staff satisfaction, compared to SOC distraction techniques.

Our results are consistent with multiple previous studies highlighting VR's role in pediatric pain management. VR has been shown to significantly reduce procedural pain in children undergoing venipuncture, burn care, and vaccinations.^32^, ^33^

The reduction in anxiety was particularly evident at all measurement time points—before, during, and after the procedure. Notably, children in the VR group exhibited lower levels of preprocedural anxiety, suggesting that anticipatory relief may contribute to the overall effect of VR. This phenomenon has also been reported in studies involving minor pediatric surgeries, reinforcing the idea that the expectation of engaging with an immersive distraction tool can help modulate emotional responses even before the procedure begins.^17^, ^34^

Pain and fear were also significantly reduced in the VR group. This is consistent with both the Gate Control Theory of Pain and the Multiple Resource Theory, which suggest that immersive environments divert cognitive attention and sensory processing resources away from nociceptive stimuli.^35^ Functional magnetic resonance imaging (fMRI) studies have demonstrated that VR can reduce activity in pain‐related brain areas such as the anterior cingulate cortex and insula, supporting the notion of VR as a psychological analgesic.^36^, ^37^

Our exploratory age‐stratified analysis indicated that the largest benefits of VR over SOC were observed in the youngest participants, with a gradual reduction in effect as age increased. This observation is in line with previous evidence showing that pain and anxiety are more pronounced in very young children.^38^ Although our study did not include children younger than 4 years, the enhanced benefit observed in the 4–5 year subgroup suggests that VR could provide even greater advantages in younger age groups. Future research should investigate its application in toddlers and preschool‐aged children, who may be particularly vulnerable to procedural distress. Although these findings should be interpreted with caution, they suggest that age may modulate the response to immersive distraction, and this factor should be considered when designing future RCTs and tailoring VR interventions to different pediatric subgroups.

Importantly, our findings also revealed a marked improvement in procedural compliance among children in the VR group. Compliance rates approached 100% under VR conditions, whereas children in the SOC group exhibited significantly more physical and emotional resistance. This difference is clinically significant, as noncompliance during allergy testing is a known cause of test failure, repeated appointments, and increased healthcare burden.^39^ Moreover, children who had a positive experience with VR showed greater willingness to participate in future medical visits, consistent with prior research.^33^, ^40^

From a physiological standpoint, parameters such as HR, BP, and SpO_2_ remained stable across both groups, indicating that VR did not elicit any adverse physiological effects. This aligns with prior studies confirming the safety of VR use in pediatric settings.^41^, ^42^

Additionally, staff members reported smoother procedural workflows, enhanced cooperation from patients, and reduced emotional strain when using VR, confirming earlier findings in pediatric emergency medicine.^43^, ^44^ VR not only alleviates child distress but also significantly reduces caregiver anxiety, fostering a more positive procedural experience for families and potentially enhancing parental trust in allergy care delivery.^45^ In addition to reducing patient distress, the implementation of VR during pediatric procedures has been shown to streamline clinical workflows by decreasing procedural delays due to emotional resistance, which can ultimately enhance clinic throughput and resource allocation.^45^

Beyond immediate clinical benefits, VR may also carry long‐term advantages. By engaging children in a controlled, multisensory, and interactive environment, VR can help reshape how they perceive and experience medical procedures. Over time, this could lead to a reduction in procedural distress through positive reinforcement, preventing the escalation of healthcare‐related anxiety.^45^, ^46^ Beyond immediate relief, repeated exposure to VR‐based medical procedures may promote positive associative learning, reducing the risk of developing procedural phobias and improving long‐term healthcare engagement in children.^46^

Another key consideration is cost‐effectiveness. Studies suggest that VR could reduce the need for pharmacological interventions like sedation, decrease retesting due to noncompliance, and improve procedural efficiency—factors that contribute to reduced healthcare costs over time.^47^, ^48^, ^49^, ^50^ While our study did not include an economic analysis, the potential for long‐term savings and improved resource utilization is noteworthy.

Despite these promising outcomes, some limitations must be acknowledged. The study was conducted at a single center and included a relatively small sample size, which may limit generalizability. Furthermore, while the crossover design controlled for many confounding factors, novelty effects and individual engagement levels with VR content may have influenced outcomes. Another important limitation is the open‐label design, which may have introduced bias in the reporting of subjective outcomes such as pain, anxiety, and fear, as well as in observer‐based ratings of compliance and staff satisfaction. Although the six‐month washout period could theoretically allow for maturation effects in younger children, our analysis did not detect significant sequence effects, supporting the robustness of our findings despite the relatively long interval. Although we used validated instruments, standardized protocols, and trained personnel to reduce this risk, and although outcome assessors were unaware of the study hypothesis, the lack of blinding could have still influenced perceptions. Future studies using blinded evaluators or objective physiological endpoints would strengthen internal validity and provide more robust evidence. Additionally, investigations exploring repeated VR exposure and age‐personalized immersive content are warranted to evaluate sustained benefits and long‐term engagement. Neuroimaging research, such as fMRI, could also help elucidate the neural mechanisms underlying VR's effect. Finally, it should be acknowledged that our study did not include a direct comparison between VR and passive screen‐based distraction techniques, such as video or cartoon viewing, which represent low‐cost and widely accessible strategies for reducing procedural distress. Previous randomized trials and meta‐analyses have shown that immersive VR generally achieves greater reductions in anxiety and pain than conventional video distraction, although considerable heterogeneity persists across studies depending on the type and duration of the procedure, patient age, and immersion level.^32^, ^47^, ^51^ While our findings confirmed the safety of VR in this context, potential adverse effects—including cybersickness, visual fatigue, or reduced situational awareness—should be acknowledged. However, given the very short duration of the SPT procedure, the risk of such events is minimal and their clinical impact negligible, as also supported by the absence of any reported adverse reactions in our study. Future research directly comparing immersive VR with standard video distraction could help further delineate their relative efficacy and cost‐effectiveness in brief pediatric procedures.

In conclusion, our study provides compelling evidence that VR significantly reduces anxiety, fear, and pain during SPT, improves procedural compliance, and enhances staff satisfaction without inducing physiological stress. These findings support the integration of VR as a standard adjunctive tool in pediatric allergy diagnostics. Broader implementation, alongside further research into long‐term outcomes, economic impact, and personalization strategies, could transform pediatric procedural care by fostering a more cooperative, efficient, and child‐friendly clinical environment.

## AUTHOR CONTRIBUTIONS

**Rita Nocerino:** Conceptualization; methodology; investigation; formal analysis; data curation; resources; project administration; writing – original draft; writing – review and editing; supervision. **Adriana Napolitano:** Validation; investigation; visualization. **Giorgio Bedogni:** Methodology; formal analysis; data curation; writing – original draft. **Teresa Rea:** Validation; investigation; supervision; writing – original draft. **Silvio Simeone:** Validation; investigation; supervision; writing – original draft. **Antonio Masino:** Investigation; validation; visualization. **Erika Caldaria:** Investigation; validation; visualization. **Serena Coppola:** Investigation; validation; visualization. **Laura Carucci:** Investigation; validation; visualization. **Roberto Berni Canani:** Methodology; investigation; visualization; resources; project administration; supervision; writing – original draft.

## CONFLICT OF INTEREST STATEMENT

Roberto Berni Canani has had the following relevant financial relationships with the following manufacturers: Biostime (research grant), Ch. Hansen (research grant, speaker), DBV (research grant), Dr. Schar (research grant), Humana (research grant), iHealth (research grant), Kraft‐Heinz (research grant, speaker), Mead Johnson Nutrition (research grant, speaker), Nestlè (research grant, speaker), Novalac (research grant, speaker), Nutricia (research grant, speaker), Sanofi (research grant, speaker) as part of publicly funded research projects with the support of the Italian Ministry of Health, the Italian Ministry of the University and Research, and the EU. The other authors declared that they have no conflicts of interest.

## PEER REVIEW

The peer review history for this article is available at https://www.webofscience.com/api/gateway/wos/peer‐review/10.1111/pai.70235.

## Data Availability

The data that support the findings of this study are available from the corresponding author upon reasonable request.
